# Intra-breath respiratory mechanics of prematurity-associated lung disease phenotypes in school-aged children

**DOI:** 10.1183/23120541.00840-2024

**Published:** 2025-03-31

**Authors:** Michael Cousins, Kylie Hart, Bence Radics, A. John Henderson, Zoltán Hantos, Peter D. Sly, Sailesh Kotecha

**Affiliations:** 1 Department of Child Health, Cardiff University School of Medicine, Cardiff, UK; 2 Department of Paediatrics, Cardiff and Vale University Health Board, Cardiff, UK; 3 Department of Pathology, University of Szeged, Szeged, Hungary; 4 MRC Integrative Epidemiology Unit, Population Health Sciences, Bristol Medical School, University of Bristol, Bristol, UK; 5 Department of Anesthesiology and Intensive Therapy, Semmelweis University, Budapest, Hungary; 6 Child Health Research Centre, The University of Queensland, South Brisbane, Australia

## Abstract

**Background:**

Intra-breath oscillometry potentially offers detailed information regarding airway function, with increasing magnitude of difference between resistance and reactance at end-expiration to end-inspiration potentially associated with obstructive airway disease, but less is known about specific respiratory mechanics in preterm-born children using this methodology. We investigated whether different spirometry phenotypes of prematurity-associated lung disease (PLD) have specific intra-breath oscillometry features.

**Methods:**

167 school-aged (7–12 years) children, 14 with prematurity-associated obstructive lung disease (POLD; forced expiratory volume in 1 s (FEV_1_) <lower limit of normal (LLN), FEV_1_/forced vital capacity (FVC) <LLN), 11 with prematurity-associated preserved ratio impaired spirometry (pPRISm; FEV_1_ <LLN, FEV_1_/FVC ≥LLN), 90 preterm controls (FEV_1_ ≥LLN) and 52 term controls, performed intra-breath oscillometry at baseline, following maximal cardiopulmonary exercise testing and following post-exercise bronchodilation.

**Results:**

Children with POLD showed greater resistance and more negative reactance throughout the respiratory cycle, including at zero-flow states of end-expiration and end-inspiration. The difference between end-expiration and end-inspiration did not show differences between groups until corrected for tidal volume, whereby children with POLD and pPRISm both demonstrated approximately two-fold greater difference compared to both preterm and term controls for resistance (2.24 and 2.22 *versus* 1.28 and 1.11 hPa·s·L^−1^, respectively), and in particular a greater magnitude of difference for reactance for children with POLD *versus* preterm and term controls only (−1.58 *versus* −0.26 and 0.03 hPa·s·L^−1^, respectively).

**Conclusions:**

Intra-breath respiratory mechanics for preterm-born children with an obstructive lung phenotype have greater impedance throughout the respiratory cycle, features different to those observed in children with other wheeze phenotypes including preschool wheeze and asthma.

## Shareable abstract

**Preterm-born children with an obstructive airway phenotype have differing intra-breath respiratory system mechanics compared to children with other obstructive airway diseases** https://bit.ly/3A5bfCu

## Introduction

Oscillometry (previously known as forced oscillation technique) is a useful tool for identifying differences in airway mechanics between populations. Changes occurring in respiratory impedance (*Z*_rs_) and its components resistance (*R*_rs_) and reactance (*X*_rs_) during tidal breathing can be beneficial for improving understanding of disease pathology, including the peripheral airway disease identified in preterm-born children [1]. Intra-breath oscillometry offers insight into dynamic changes that occur throughout the respiratory cycle [2, 3]. This information may be able to further differentiate between pathological entities [4]. This method superimposes a single frequency wave on tidal breathing and assesses changes in impedance at different points of the respiratory cycle, in particular those relating to zero-flow states, *i.e.* end-expiration and end-inspiration.

Intra-breath oscillometry has been used in a range of ages including in infancy [5], where the difference between end-expiration and end-inspiration respiratory system resistance (Δ*R*) and reactance (Δ*X*) was predictive for lower respiratory tract infection, potentially a result of airway flow abnormalities which would not be clinically detectable [6]. Similarly, detection of airway obstruction presenting clinically as preschool wheeze or asthma is potentially identifiable with increasing magnitude of Δ*R*, with a Δ*R* of ≥1.42 hPa·s·L^−1^ able to distinguish between children with recurrent wheeze episodes and healthy controls [2]. This suggests that in preschool wheeze/asthma phenotypes of obstructive lung disease there is a predilection to the expiratory component of the respiratory cycle being affected. Intra-breath oscillometry has also been used in adults with obstructive airway disease including COPD [3] and asthma [7], with similar increasing magnitude for Δ*R* and Δ*X* in the latter, and flow limitation identified on volume *versus* reactance loops in the former.

Preterm-born children are known to have disrupted lung growth [8] and are at risk of long-term lung dysfunction. Preterm-born populations have increased respiratory symptoms including wheezing [9], spirometry abnormalities [10] (including similar dysfunction noted over time [11]) and exercise impairment [12, 13]. We have shown increasing evidence that prematurity-associated lung disease (PLD) does not fall into a single pathological entity, but more likely differing phenotypes within the preterm-born population [13–15], including prematurity-associated obstructive lung disease (POLD) and prematurity-associated preserved ratio impaired spirometry (pPRISm). PRISm in adult populations has been shown to be associated with COPD and all-cause mortality [16, 17]; however, less is known about its significance when identified in childhood, including in preterm populations.

In children, oscillometry has an advantage over other, effort-dependent, lung function tests such as spirometry, as it is only reliant on tidal breathing, and has been demonstrated to be feasible in infant [6] and preschool [18] age groups. Additionally, with higher rates of neurodisability in children born preterm [19], and the associated difficulty with performing spirometry in such patients [20], oscillometry is particularly suited for preterm-born children.

The use of intra-breath oscillometry in preterm populations has been relatively limited so far. A small sample of largely late preterm-born children identified small but significantly greater magnitudes of Δ*R* and Δ*X* compared to full-term controls [21]. Given the overlap of potential pathology, *i.e.* airway obstruction, between preterm-associated lung disease and wheeze or asthma, it would be reasonable to hypothesise that similar Δ*R* and Δ*X* changes may be identifiable in preterm-born children with an obstructive phenotype. Thus, we compared intra-breath oscillometry data between three phenotypes of preterm children based on spirometric outcomes (POLD, pPRISm and preterm controls (PT_c_)) and a control group of term-born children (T_c_), with additional measurements taken at post-exercise and post-exercise bronchodilation time-points.

## Methods

### Population, spirometry and exercise testing

Preterm- and term-born children from birth years 2005 to 2011, identified during a previous questionnaire study [9, 22], were prospectively recruited for the Respiratory Health Outcomes in Neonates (RHiNO) study (EudraCT: 2015-003712-20) as previously described [13, 15, 23]. Following screening, where spirometry was performed by trained research nurses, children meeting the inclusion criteria (gestational age at birth ≤34 weeks gestation for preterm-born children and ≥37 weeks gestation for term-born children; age 7–12 years; geographically accessible) were invited for in-depth lung function testing including spirometry, exercise testing and oscillometry at the Noah's Ark Children's Hospital for Wales (Cardiff, UK), from January 2017 to August 2019. All preterm-born children with forced expiratory volume in 1 s (FEV_1_) ≤85% predicted at screening were invited, so they could participate in the randomised control trial [23], together with the first 10 preterm-born children with FEV_1_ >85% predicted as controls during each calendar month. Term-born children with FEV_1_ >90% predicted were randomly invited to participate. Children who could not perform acceptable spirometry did not complete the full visit. Children with significant congenital/cardiac/neurodevelopmental abnormalities were excluded and testing was postponed in children with a recent (within the past 3 weeks) respiratory tract infection.

Spirometry and exercise testing have been described elsewhere in greater detail [13]. Briefly, spirometry was performed in line with American Thoracic Society/European Respiratory Society guidance [24] using the MasterScreen Body/PFT systems with SentrySuite measurement software version 2.17 (Vyaire Medical, Höchberg, Germany). Global Lung Function Initiative (GLI) equations were used as reference standards for spirometry values [25].

Spirometry was used to classify children into the following phenotypes of interest as previously described [15]: POLD: FEV_1_ <LLN, FEV_1_/forced vital capacity (FVC) <lower limit of normal (LLN); pPRISm: FEV_1_ <LLN, FEV_1_/FVC ≥LLN; PT_c_: FEV_1_ ≥LLN; and T_c_: FEV_1_ >90% predicted.

Cardiopulmonary exercise testing was performed on a Pediatric Cycle Ergometer (Lode, Groningen, The Netherlands) with a MasterScreen CPX system (Vyaire Medical). “Maximal” testing was achieved if at least two of the following criteria were met: respiratory exchange ratio >1.00; heart rate ≥80% predicted (220 beats·min^−1^ minus age); ≥9/10 on OMNI scale (pictorial scale for rating of perceived exertion [26]); and oxygen uptake plateau reached.

#### Oscillometry

Oscillometry was performed using a custom-built loudspeaker-in-box device, designed to operate during post-exercise rapid breathing, as previously described (see details in the supplementary material) [1].

A noseclip was worn and cheeks firmly held during testing. The loudspeaker superimposed a 10 Hz soundwave at 0.1 s intervals onto tidal breathing, with respiratory impedance measured at the mouth using pressure and flow sensors. A minimum of three recordings lasting 23.5 s were obtained and analysis performed on the recording with most regular artefact-free breaths (*i.e.* no coughs, glottic closure or breath holds). Average measures across breaths for key parameters were calculated, for mean impedance (reactance (*R*) and reactance (*X*)) measured at end-expiration (eE)/inspiration (eI), and mean impedance during expiration (meanE) and inspiration (meanI) calculated.

Intra-breath oscillometry measures were obtained at baseline, 20 min post-maximal exercise testing and following administration of post-exercise bronchodilator (400 μg salbutamol (Salamol; Teva, Castleford, UK) administered with a metered-dose inhaler using a Volumatic spacer (GSK, Brentford, UK)).

### Ethical approval

Parents and children provided informed written consent or assent, respectively, with ethical approval granted by the Southwest Central Bristol Ethics Committee (15/SW/0289).

### Statistical analysis

One-way ANOVA with Bonferroni correction was used for multigroup comparisons for continuous data. Categorical data were assessed using Pearson's Chi-squared test. Two-way repeated measures ANOVA with Bonferroni correction was used for within-group and between-group comparisons across time-points. A p-value <0.05 was considered statistically significant. Where data were missing at one or more time-points (recording issue, time constraint, test quality or participant declining test), all data for these participants were excluded from the repeated measures analysis. Statistical analysis was performed using SPSS Statistics version 26 (IBM, Armonk, NY, USA).

## Results

### Participant details

**FIGURE 1:**
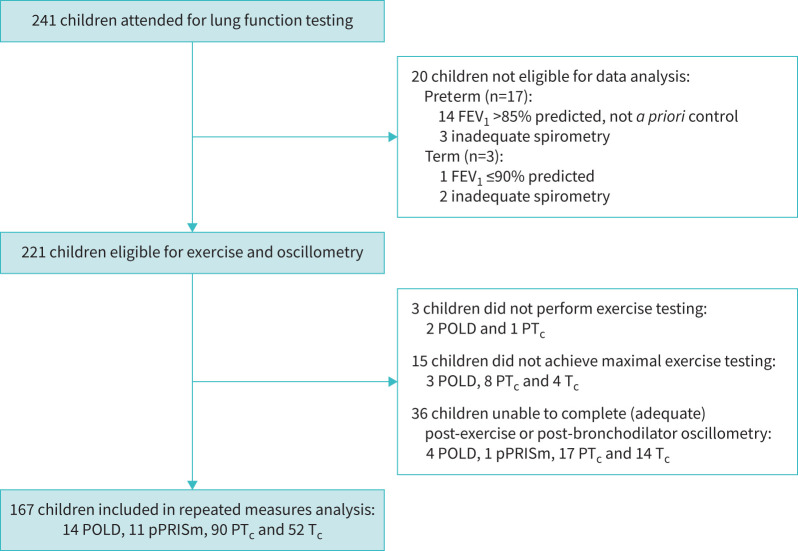
Recruitment flow diagram displaying number of participants performing lung function testing and those included in the final analysis. POLD: prematurity-associated obstructive lung disease; pPRISm: prematurity-associated preserved ratio impaired spirometry; PT_c_: preterm control group; T_c_: term control group; FEV_1_: forced expiratory volume in 1 s.

Of 241 original invited participants, 20 were excluded due to inadequate spirometry (figure 1). Three children did not perform exercise testing and 15 children did not achieve maximal exercise testing, and thus were excluded from full analysis. Of the remaining 203 children, 36 had one or more time-points missing from their oscillometry testing (missed or declined test, suboptimal quality of recording or recording issue). 167 children were included in repeated measures analysis of oscillometry data and were phenotyped based on their spirometry into the following groups: 14 POLD, 11 pPRISm, 90 PT_c_ and 52 T_c_.

Participant demographics are summarised in table 1. Anthropometric measurements were similar between groups with the exception of lower weight z-scores in children with pPRISm compared to both control groups. There were no differences in raw or z-score heights between the groups. The PT_c_ group was slightly older than the T_c_ group. Children with POLD were born at an earlier gestation compared to the PT_c_ group (29.3 *versus* 31.1 weeks gestation). There were no differences for birthweight, invasive ventilation or chronic lung disease of prematurity rates between the preterm groups. Children with POLD had higher rates compared to T_c_ children for wheeze ever (86% *versus* 25%; and *versus* 46% in PT_c_), recent (last 12 months) wheeze (50% *versus* 15%), asthma diagnosis (43% *versus* 10%) and salbutamol use (36% *versus* 8%). There was no difference in rates of exposure to maternal smoking.

**TABLE 1:** Baseline characteristics of participants including anthropometric, perinatal and respiratory details for prematurity-associated obstructive lung disease (POLD), prematurity-associated preserved ratio impaired spirometry (pPRISm), preterm (PT_c_) and term (T_c_) control groups

	POLD (n=14)	pPRISm (n=11)	PT_c_ (n=90)	T_c_ (n=52)
**Current demographics**				
Age, years	11 (10.2–11.7)	11.1 (10.2–12.1)	**11.2 (11–11.4)** ^+ +^	10.5 (10.2–10.8)
Male	9 (64)	2 (18)	46 (51)	27 (52)
Height, cm	142.5 (136.8–148.2)	142.8 (133.3–152.3)	146.8 (144.9–148.6)	143.4 (140.8–145.9)
Height, z-score	−0.13 (−0.7–0.43)	−0.32 (−1.35–0.71)	0.26 (0.09–0.43)	0.4 (0.11–0.69)
Weight, kg	37.9 (32.5–43.3)	35.5 (26.5–44.6)	40 (37.9–42.1)	36.9 (34.7–39)
Weight, z-score	0.21 (−0.43–0.85)	**−0.63 (−1.84–0.58)** ^#,¶^	0.32 (0.11–0.54)	0.39 (0.12–0.66)
BMI, kg·m^−2^	18.5 (16.6–20.3)	16.8 (14.4–19.2)	18.4 (17.6–19.1)	17.8 (17.1–18.5)
BMI, z-score	0.36 (−0.37–1.08)	−0.67 (−1.74–0.39)	0.18 (−0.08–0.45)	0.26 (−0.02–0.54)
**Perinatal demographics**				
Gestation, decimal weeks	**29.3 (27.6–31.0)** ^†,‡‡‡^	**30.0 (28.0–32.0)** ^¶¶¶^	**31.1 (30.5–31.7)** ^+ + +^	40.0 (39.7–40.3)
Birthweight, g	**1361 (1063–1660)** ^‡‡‡^	**1487 (1077–1898)** ^¶¶¶^	**1721 (1602–1840)** ^+ + +^	3490 (3363–3617)
Birthweight, z-score	−0.07 (−0.63–0.48)	0.03 (−0.73–0.78)	0.22 (−0.07–0.52)	0.03 (−0.21–0.27)
IUGR	1 (7)	2 (18)	14 (16)	2 (4)
Antenatal steroids	**12 (86)** ^‡‡‡^	**10 (91)** ^¶¶¶^	**74 (82)** ^+ + +^	0 (0)
Invasive ventilation	**9 (64)** ^‡‡‡^	**4 (36)** ^¶¶¶^	**33 (37)** ^+ + +^	0 (0)
CLD of prematurity	**5 (36)** ^‡‡‡^	**3 (27)** ^¶¶¶^	**19 (21)** ^+ + +^	0 (0)
**Respiratory history**				
Doctor-diagnosed asthma	**6 (43)** ^‡^	2 (18)	20 (22)	5 (10)
Wheeze ever	**12 (86)** ^†,‡‡‡^	6 (55)	41 (46)	13 (25)
Recent wheeze	**7 (50)** ^‡^	2 (18)	19 (21)	8 (15)
Current salbutamol use	**5 (36)** ^‡^	1 (9)	16 (18)	4 (8)
Current maternal smoking	1 (7)	1 (9)	6 (7)	0 (0)

Data are presented as mean (95% confidence interval) for continuous data (one-way ANOVA with Bonferroni correction) or n (%) for categorical data (Pearson's Chi-squared test). BMI: body mass index; IUGR: intrauterine growth restriction; CLD: chronic lung disease. Significance symbols for bold data values: *: POLD *versus* pPRISm; ^†^: POLD *versus* PT_c_; ^‡^: POLD *versus* T_c_; ^#^: pPRISm *versus* PT_c_; ^¶^: PRISm *versus* T_c_; ^+^: PT_c_ *versus* T_c_. Single symbol: p<0.05; double symbol: p<0.01; triple symbol: p<0.001.

### Respiratory parameters

Table 2 summarises the participants’ respiratory parameters. Expiratory time (*T*_E_) between groups showed no differences at any time-point. However, on repeated measures across time-points, there was a small decrease in expiratory time for preterm and term controls from baseline to post-exercise (both 1.7 to 1.5 s) and for children with POLD from post-exercise to post-exercise bronchodilation time-points (1.6 to 1.3 s). The POLD group had a higher baseline proportion of expiratory time to total respiratory time (*T*_E_/*T*_tot_) for the POLD group compared to both preterm and term controls (0.55 *versus* 0.52 for both controls). This difference persisted to the post-exercise time-point but not to post-exercise bronchodilation. Respiratory rate (*F*_br_) increased in preterm and term controls post-exercise (20.5 to 22.6 and 20.2 to 22.9 breaths·min^−1^ from baseline to post-exercise, respectively). Repeated measures showed no significant changes at the post-exercise bronchodilation time-point.

**TABLE 2:** Respiratory parameters for prematurity-associated obstructive lung disease (POLD), prematurity-associated preserved ratio impaired spirometry (pPRISm), preterm (PT_c_) and term (T_c_) control groups, at baseline, at post-exercise and at post-exercise bronchodilation (BD) time-points

	POLD (n=14)	pPRISm (n=11)	PT_c_ (n=90)	T_c_ (n=52)
***T*_E_ (s)**				
Baseline	1.5 (1.3–1.8)	1.6 (1.4–1.9)	1.7 (1.6–1.8)	1.7 (1.5–1.9)
Post-exercise	1.6 (1.2–2)	1.5 (1.1–1.9)	^§^ 1.5 (1.4–1.6)	^§§^ 1.5 (1.4–1.6)
Post-exercise BD	^ƒ^ 1.3 (1.0–1.6)	1.4 (1.1–1.7)	1.5 (1.4–1.6)	1.5 (1.4–1.6)
***T*_E_/*T*_tot_**				
Baseline	**0.55 (0.52–0.57)** ^†^	0.53 (0.51–0.56)	0.52 (0.51–0.53)	0.52 (0.51–0.53)
Post-exercise	**0.55 (0.52–0.58)** ^†,‡^	0.51 (0.49–0.53)	0.52 (0.51–0.53)	0.52 (0.51–0.53)
Post-exercise BD	0.53 (0.49–0.57)	0.52 (0.50–0.54)	0.53 (0.52–0.54)	0.53 (0.52–0.54)
***T*_PEF_/*T*_E_**				
Baseline	0.28 (0.24–0.32)	0.37 (0.28–0.47)	0.33 (0.31–0.35)	0.34 (0.31–0.36)
Post-exercise	**0.26 (0.22–0.31)** ^†,‡^	0.34 (0.26–0.42)	0.34 (0.32–0.36)	0.34 (0.32–0.37)
Post-exercise BD	0.35 (0.25–0.44)	0.39 (0.32–0.47)	0.35 (0.33–0.38)	0.35 (0.32–0.38)
***V*_T_ (L)**				
Baseline	0.48 (0.37–0.59)	0.44 (0.32–0.55)	0.58 (0.53–0.63)	0.60 (0.52–0.68)
Post-exercise	0.53 (0.41–0.66)	0.38 (0.33–0.43)	0.55 (0.51–0.59)	0.57 (0.49–0.66)
Post-exercise BD	0.55 (0.41–0.68)	0.44 (0.36–0.51)	0.58 (0.53–0.63)	0.59 (0.53–0.66)
***F*_br_ (breaths·min^−1^)**				
Baseline	23.4 (19.6–27.3)	20.9 (17–24.8)	20.5 (19.3–21.7)	20.2 (18.4–22.0)
Post-exercise	23.8 (19.3–28.3)	24.5 (18.4–30.5)	^§^ 22.6 (21.2–24.0)	^§^ 22.9 (20.9–24.8)
Post-exercise BD	26.5 (22.4–30.6)	25.8 (20.0–31.6)	23.7 (21.9–25.4)	22.5 (20.9–24.1)

Data are presented as mean (95% confidence interval) for continuous data (two-way ANOVA with Bonferroni correction). *T*_E_: expiratory time; *T*_tot_: total expiratory time; *T*_PEF_: time to peak expiratory flow; *V*_T_: tidal volume; *F*_br_: breathing frequency. Significance symbols for bold data values: *: POLD *versus* pPRISm; ^†^: POLD *versus* PT_c_; ^‡^: POLD *versus* T_c_; ^#^: pPRISm *versus* PT_c_; ^¶^: PRISm *versus* T_c_; ^+^: PT_c_ *versus* T_c_. Significance symbols for underlined data values: ^§^: baseline *versus* post-exercise; ^ƒ^: post-exercise *versus* post-BD. Single symbol: p<0.05; double symbol: p<0.01.

### End-respiratory impedance

Table 3 summarises the participants’ end-respiratory impedance. Evaluation of resistance at end-expiration (*R*_eE_) and end-inspiration (*R*_eI_) at baseline revealed higher resistance in the POLD group compared to both preterm and term control groups (*R*_eE_ 6.7 *versus* 5.2 *versus* 5.1 hPa·s·L^−1^ and *R*_eI_ 5.8 *versus* 4.5 *versus* 4.4 hPa·s·L^−1^); however, no difference was observed in the difference between these two values between any groups at baseline (Δ*R* 0.9 *versus* 1.0 *versus* 0.7 *versus* 0.7 hPa·s·L^−1^). When standardised against the change in tidal volume (Δ*R*/*V*_T_), there was a non-significant trend towards higher values in POLD and pPRISm groups (2.24 and 2.22 *versus* 1.28 and 1.11 hPa·s·L^−2^ greater than PT_c_ and T_c_, respectively).

**TABLE 3:** End-respiratory (zero-flow) impedance for prematurity-associated obstructive lung disease (POLD), prematurity-associated preserved ratio impaired spirometry (pPRISm), preterm (PT_c_) and term (T_c_) control groups, at baseline, at post-exercise and at post-exercise bronchodilation (BD) time-points

	POLD (n=14)	pPRISm (n=11)	PT_c_ (n=90)	T_c_ (n=52)
***R*_eE_ (hPa·s·L^−1^)**				
Baseline	**6.7 (5.6–7.9)** ^†,‡‡^	6.3 (5.2–7.3)	5.2 (4.9–5.6)	5.1 (4.7–5.5)
Post-exercise	**7.2 (6.1–8.3)** ^†††,‡‡^	6 (4.6–7.4)	5.2 (4.9–5.6)	5.3 (4.9–5.8)
Post-exercise BD	^ƒƒƒ^ 4.7 (4.0–5.4)	^ƒƒ^ 5.1 (3.8–6.4)	^ƒƒƒ^ 4.3 (4–4.7)	^ƒƒƒ^ 4.5 (4.1–4.9)
***R*_eI_ (hPa·s·L^−1^)**				
Baseline	**5.8 (4.9–6.7)** ^††,‡‡^	5.3 (4.5–6)	4.5 (4.2–4.8)	4.4 (4.1–4.8)
Post-exercise	**6.2 (5.4–7.1)** ^†††,‡‡^	5.6 (4.6–6.6)	4.6 (4.4–4.9)	4.7 (4.3–5.1)
Post-exercise BD	^ƒƒƒ^ 4.4 (3.8–4.9)	^ƒƒƒ^ 4.6 (3.6–5.5)	^ƒƒƒ^ 3.8 (3.5–4)	^ƒƒƒ^ 4 (3.7–4.3)
**Δ*R* (hPa·s·L^−1^)**				
Baseline	0.9 (0.4–1.4)	1.0 (0.4–1.6)	0.7 (0.6–0.9)	0.7 (0.5–0.9)
Post-exercise	0.9 (0.3–1.5)	^§^ 0.4 (−0.2–1)	0.6 (0.5–0.8)	0.6 (0.4–0.9)
Post-exercise BD	^ƒ^ 0.3 (0.0–0.7)	0.5 (−0.1–1.1)	0.6 (0.4–0.8)	0.5 (0.3–0.8)
**Δ*R*/*V*_T_ (hPa·s L^−2^)**				
Baseline	2.24 (1.12–3.36)	2.22 (0.94–3.51)	1.28 (1–1.55)	1.11 (0.68–1.55)
Post-exercise	1.90 (0.63–3.18)	1.08 (−0.55–2.71)	1.08 (0.82–1.35)	0.96 (0.55–1.37)
Post-exercise BD	^ƒ^ 0.96 (0.10–1.81)	1.2 (−0.19–2.59)	1.00 (0.67–1.32)	0.84 (0.46–1.23)
***X*_eE_ (hPa·s·L^−1^)**				
Baseline	**−3.2 (−4.2– −2.1)** ^†††,‡‡‡^	−1.7 (−2.5– −0.8)	−1.2 (−1.6– −0.9)	−0.9 (−1.1– −0.7)
Post-exercise	**−3.4 (−4.0– −2.7)** ^†††,‡‡‡^	−2.1 (−3.5– −0.8)	−1.3 (−1.6– −1)	−1.2 (−1.5– −0.9)
Post-exercise BD	^ƒƒƒ^ −1.1 (−1.6– −0.7)	^ƒƒ^ −1.2 (−2.1– −0.3)	^ƒƒƒ^ −0.7 (−0.9– −0.5)	^ƒƒ^ −0.9 (−1.1– −0.6)
***X*_eI_ (hPa·s·L^−1^)**				
Baseline	−2.5 (−3.2– −1.8)* ^,†††,‡‡‡^	−1.5 (−2.1– −1)	−1.1 (−1.3– −0.9)	−0.9 (−1– −0.7)
Post-exercise	**−2.8 (−3.2– −2.4)** ^†††,‡‡‡^	^§§^ **−2.2 (−3– −1.3)** ^##,¶¶^	−1.2 (−1.4– −1)	^§^ −1.1 (−1.4– −0.9)
Post-exercise BD	^ƒƒƒ^ −1.2 (−1.5– −0.8)	^ƒƒƒ^ −1.4 (−2.1– −0.6)	^ƒƒƒ^ −0.7 (−0.9– −0.6)	^ƒƒ^ −0.9 (−1.1– −0.7)
**Δ*X* (hPa·s·L^−1^)**				
Baseline	−0.7 (−1.1– −0.2)	−0.1 (−0.7–0.4)	−0.2 (−0.4–0.0)	0.0 (−0.2–0.1)
Post-exercise	−0.6 (−1.0– −0.2)	0.0 (−0.8–0.8)	−0.1 (−0.2–0.1)	−0.1 (−0.3–0.2)
Post-exercise BD	^ƒƒ^ 0.0 (−0.3–0.4)	0.2 (−0.1–0.5)	0.0 (−0.1–0.2)	0.0 (−0.1–0.2)
**Δ*X*/*V*_T_ (hPa·s L^−2^)**				
Baseline	**−1.58 (−2.63– −0.52)** ^†,‡‡^	0.02 (−1.17–1.2)	−0.26 (−0.61–0.1)	0.03 (−0.24–0.31)
Post-exercise	**−1.14 (−1.89– −0.40)** ^‡^	0.20 (−1.73–2.12)	−0.05 (−0.34–0.24)	0.14 (−0.28–0.56)
Post-exercise BD	^ƒƒ^ 0.03 (−0.54–0.61)	0.57 (−0.23–1.37)	0.09 (−0.17–0.34)	0.17 (−0.11–0.44)

Data are presented as mean (95% confidence interval) for continuous data (two-way ANOVA with Bonferroni correction). *R*: resistance; *X*: reactance; e: end-respiratory; E: expiration; I: inspiration; Δ: difference; *V*_T_: tidal volume. Significance symbols for bold data values: *: POLD *versus* pPRISm; ^†^: POLD *versus* PT_c_; ^‡^: POLD *versus* T_c_; ^#^: pPRISm *versus* PT_c_; ^¶^: PRISm *versus* T_c_; ^+^: PT_c_ *versus* T_c_. Significance symbols for underlined data values: ^§^: baseline *versus* post-exercise; ^ƒ^: post-exercise *versus* post-BD. Single symbol: p<0.05; double symbol: p<0.01; triple symbol: p<0.001.

Repeated measures analysis showed no difference for end-expiratory or end-inspiratory resistance for any group from baseline to post-exercise. All groups demonstrated a reduction in end-expiratory and end-inspiratory resistance from post-exercise to post-exercise bronchodilation. At the post-exercise bronchodilation time-point, no difference between children with POLD and controls remained, suggesting a greater improvement for children with POLD to bronchodilator compared to controls.

Δ*R* reduced in the pPRISm group from baseline to post-exercise (1.0 to 0.4 hPa·s·L^−1^). Δ*R* reduced in the POLD group from post-exercise to post-bronchodilation (0.9 to 0.3 hPa·s·L^−1^), and children with POLD had a reduction in Δ*R*/*V*_T_ (1.90 to 0.96 hPa·s·L^−2^) from post-exercise to post-exercise bronchodilation, a reduction not seen in any of the other groups.

End-expiratory and end-inspiratory reactances (*X*_eE_ and *X*_eI_) were significantly more negative (worse) for children with POLD when compared to both preterm and term control groups (*X*_eE_ −3.2 *versus* −1.2 *versus* −0.9 hPa·s·L^−1^ and *X*_eI_ −2.5 *versus* −1.1 *versus* −0.9 hPa·s·L^−1^). Similar to resistance, Δ*X* did not show any between-group differences, until standardised against change in tidal volume (Δ*X*/*V*_T_ −1.58 *versus* −0.26 *versus* 0.03 hPa·s·L^−2^ for POLD, PT_c_ and T_c_ groups, respectively).

Following exercise, there was no change for any group in *X*_eE_, Δ*X* and Δ*X*/*V*_T_, but there was a more negative *X*_eI_ for pPRISm children (−1.5 to −2.2 hPa·s·L^−1^).

All four groups had improved (less negative) reactance for *X*_eE_ and *X*_eI_ following post-exercise bronchodilation, but a statistically significant improvement for Δ*X* (−0.6 to 0.0 hPa·s·L^−1^) and Δ*X*/*V*_T_ (−1.14 to 0.03 hPa·s·L^−2^) was only observed for children with POLD.

**FIGURE 2:**
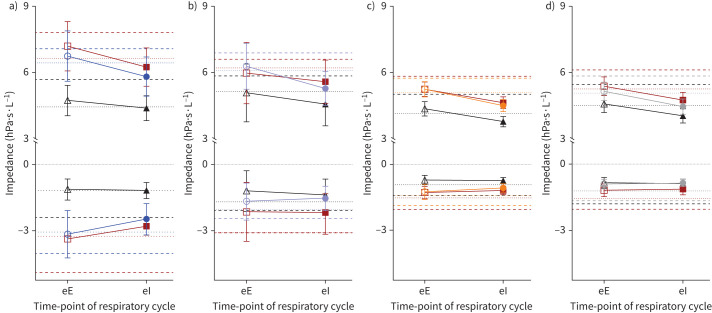
Illustration of impedance (resistance (*R*) and reactance (*X*)) at end-expiration (*R*_eE_ (top) and *X*_eE_ (bottom): open symbols), end-inspiration (*R*_eI_ (top) and *X*_eI_ (bottom): filled symbols) and mean inspiratory (dotted lines) and expiratory (dashed lines) impedances, at baseline (coloured circles), post-exercise (maroon squares) and post-exercise bronchodilation (black triangles), for a) prematurity-associated obstructive lung disease (POLD), b) prematurity-associated preserved ratio impaired spirometry (pPRISm), c) preterm controls (PT_c_) and d) term controls (T_c_). Error bars indicate 95% confidence intervals.

Figure 2 displays the end-respiratory and mean impedances at various parts of the respiratory cycle.

### Impedance loops

**FIGURE 3:**
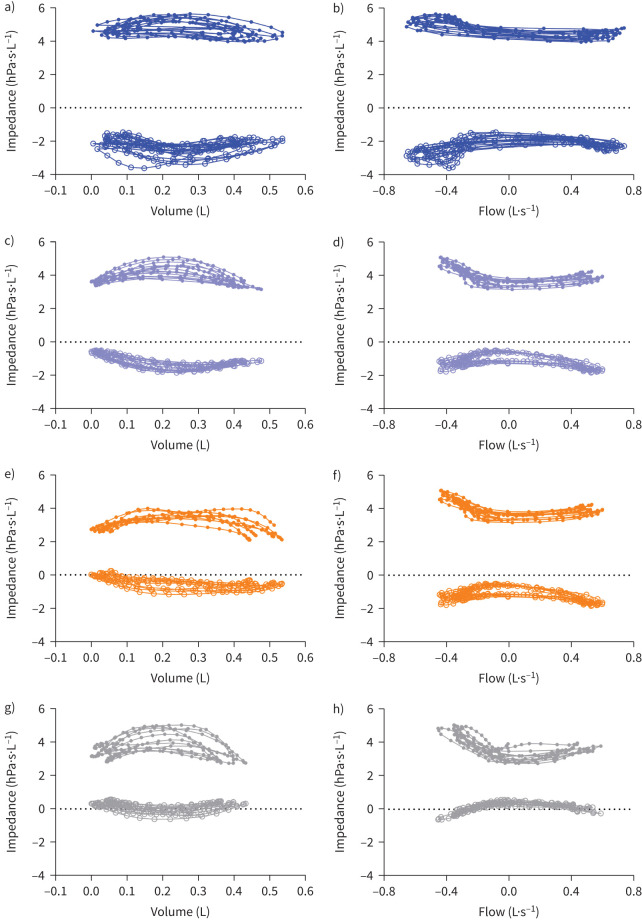
Examples of baseline a, c, e, g) impedance–volume and b, d, f, h) impedance–flow loops for a, b) prematurity-associated obstructive lung disease (POLD), c, d) prematurity-associated preserved ratio impaired spirometry (pPRISm), e, f) preterm controls (PT_c_) and g, h) term controls (T_c_).

There were no baseline differences between groups for area within either resistance–volume (*ARV*) or resistance–flow (*ARV*′) loops (table 4 and figure 3). Following exercise there was a significant increase in *ARV* for children with POLD (−0.26 to −0.70 hPa·s) which persisted after bronchodilator therapy. The preterm control children were the only group showing an increase in *ARV* after post-exercise bronchodilation (−0.48 to −0.61 hPa·s).

**TABLE 4:** Area within the impedance–volume and impedance–flow loops for prematurity-associated obstructive lung disease (POLD), prematurity-associated preserved ratio impaired spirometry (pPRISm), preterm (PT_c_) and term (T_c_) control groups, at baseline, at post-exercise and at post-exercise bronchodilation (BD) time-points

	POLD (n=14)	pPRISm (n=11)	PT_c_ (n=90)	T_c_ (n=52)
***ARV* (hPa**·**s)**				
Baseline	−0.26 (−0.44– −0.07)	−0.34 (−0.47– −0.20)	−0.42 (−0.49– −0.35)	−0.55 (−0.72– −0.39)
Post-exercise	^§^ −0.70 (−1.10– −0.31)	−0.17 (−0.32– −0.02)	−0.48 (−0.58– −0.37)	−0.56 (−0.74– −0.38)
Post-exercise BD	−0.77 (−1.23– −0.31)	−0.38 (−0.65– −0.12)	^ƒƒ^ −0.61 (−0.76– −0.45)	−0.69 (−0.89– −0.50)
***ARV*′ (hPa)**				
Baseline	2.12 (1.47–2.76)	1.81 (0.86–2.75)	1.85 (1.54–2.16)	1.76 (1.34–2.19)
Post-exercise	2.42 (1.78–3.07)	1.50 (0.33–2.67)	1.82 (1.57–2.07)	2.09 (1.57–2.62)
Post-exercise BD	1.93 (1.29–2.56)	1.84 (0.46–3.23)	1.80 (1.47–2.12)	1.90 (1.42–2.38)
***AXV* (hPa**·**s)**				
Baseline	0.49 (0.19–0.78)	0.23 (0.08–0.38)	0.28 (0.18–0.37)	0.34 (0.19–0.49)
Post-exercise	^§§^1.02 (0.42–1.61)**^,††,‡^	−0.01 (−0.34–0.32)	0.34 (0.20–0.49)	0.37 (0.20–0.54)
Post-exercise BD	0.76 (0.27–1.26)	0.23 (0.00–0.47)	0.36 (0.22–0.49)	0.44 (0.29–0.58)
***AXV*′ (hPa)**				
Baseline	−1.55 (−2.36– −0.75)	−0.64 (−1.29–0.02)	−0.61 (−0.97– −0.26)	−0.44 (−0.67– −0.21)
Post-exercise	−**1.72 (−2.40– −1.04)**^†,‡^	−0.66 (−1.92–0.60)	−0.55 (−0.79– −0.31)	−0.66 (−1.05– −0.26)
Post-exercise BD	^ƒƒ^ −0.81 (−1.45– −0.16)	−0.26 (−0.82–0.29)	^ƒƒ^ −0.29 (−0.48– −0.09)	−0.41 (−0.70– −0.12)

Data are presented as mean (95% confidence interval) for continuous data (two-way ANOVA with Bonferroni correction). *ARV*: area within resistance–volume loop; *ARV*′: area within resistance–flow loop, *AXV*: area within reactance–volume loop; *AXV*′: area within reactance–flow loop. Significance symbols for bold data values: *: POLD *versus* pPRISm; ^†^: POLD *versus* PT_c_; ^‡^: POLD *versus* T_c_; ^#^: pPRISm *versus* PT_c_; ^¶^: PRISm *versus* T_c_; ^+^: PT_c_ *versus* T_c_. Significance symbols for underlined data values: ^§^: baseline *versus* post-exercise; ^ƒ^: post-exercise *versus* post-BD. Single symbol: p<0.05; double symbol: p<0.01.

Similarly, there were no between-group differences within either reactance–volume (*AXV*) or reactance–flow (*AXV*′) loops at baseline. In the POLD group, following exercise, there was an increase in *AXV* (0.49 to 1.02 hPa·s; statistically significant) and in *AXV*′ (−1.55 to −1.72 hPa; not statistically significant), resulting in POLD having significantly greater post-exercise *AXV* compared to all three groups (1.02 *versus* −0.01 *versus* 0.34 *versus* 0.37 hPa·s) and *AXV*′ against PT_c_ and T_c_ (−1.72 *versus* −0.55 *versus* −0.66 hPa). POLD and PT_c_ groups showed a decrease in *AXV*′ following post-exercise bronchodilation (−1.72 to −0.81 and −0.55 to −0.29 hPa, respectively).

### Mean impedance

Mean resistance at baseline during expiration (*R*_meanE_) was not statistically significantly higher in the POLD group than the other groups; however, during inspiration (*R*_meanI_) it was higher compared to the PT_c_ and T_c_ groups (6.4 *versus* 5.1 *versus* 5.0 hPa·s·L^−1^, respectively) (table 5). Following exercise, the difference between resistance during expiration and inspiration (Δ*R*_mean_) in the POLD group doubled, *i.e.* increased disproportionally in expiration compared to inspiration. Almost all groups showed improvement following bronchodilation for both *R*_meanE_ and *R*_meanI_.

**TABLE 5:** Mean impedance in expiration and inspiration for prematurity-associated obstructive lung disease (POLD), prematurity-associated preserved ratio impaired spirometry (pPRISm), preterm (PT_c_) and term (T_c_) control groups, at baseline, at post-exercise and at post-exercise bronchodilation (BD) time-points

	POLD (n=14)	pPRISm (n=11)	PT_c_ (n=90)	T_c_ (n=52)
***R*_meanE_ (hPa·s·L^−1^)**				
Baseline	7.1 (5.8–8.3)	6.9 (5.9–7.9)	5.7 (5.4–6.1)	5.8 (5.3–6.3)
Post-exercise	**7.8 (6.4–9.2)** ^††,‡^	6.6 (5.3–7.9)	5.8 (5.5–6.2)	6.1 (5.6–6.6)
Post-exercise BD	^ƒƒƒ^ 5.7 (4.6–6.8)	5.8 (4.7–7.0)	^ƒƒƒ^ 5.0 (4.7–5.4)	^ƒƒƒ^ 5.4 (4.9–5.9)
***R*_meanI_ (hPa·s·L^−1^)**				
Baseline	**6.4 (5.5–7.3)** ^†,‡^	6.1 (5.2–7)	5.1 (4.8–5.4)	5.0 (4.6–5.4)
Post-exercise	**6.6 (5.7–7.6)** ^††,‡‡^	6.2 (5.0–7.5)	5.1 (4.8–5.4)	5.2 (4.8–5.6)
Post-exercise BD	^ƒƒƒ^ 4.4 (3.9–5.0)	^ƒ^ 5.1 (3.9–6.4)	^ƒƒƒ^ 4.1 (3.9–4.4)	^ƒƒƒ^ 4.4 (4.0–4.9)
**Δ*R*_mean_ (hPa·s·L^−1^)**				
Baseline	0.6 (0.2–1.1)	0.8 (0.5–1.1)	0.7 (0.5–0.8)	0.8 (0.6–1.0)
Post-exercise	^§§^ 1.2 (0.5–1.9)	0.4 (0.0–0.8)	0.8 (0.6–0.9)	0.9 (0.6–1.1)
Post-exercise BD	1.2 (0.5–2.0)	0.7 (0.3–1.1)	0.9 (0.7–1.1)	1.0 (0.7–1.2)
***X*_meanE_ (hPa·s·L^−1^)**				
Baseline	**−4.0 (−5.3– −2.8)** ^†††,‡‡‡^	−2.4 (−3.2– −1.6)	−1.9 (−2.2– −1.5)	−1.7 (−2.0– −1.3)
Post-exercise	−4.9 (−6.0– −3.8)*^,†††,‡‡‡^	−3.1 (−4.6– −1.6)	−2 (−2.4– −1.7)	−2.1 (−2.5– −1.7)
Post-exercise BD	^ƒƒƒ^ −2.4 (−3.4– −1.4)	^ƒƒ^ −2.1 (−3.1– −1)	^ƒƒƒ^ −1.4 (−1.7– −1.1)	−1.8 (−2.2– −1.4)
***X*_meanI_ (hPa·s·L^−1^)**				
Baseline	**−3.1 (−3.7– −2.4)** ^†††,‡‡‡^	−2.0 (−2.7– −1.3)	−1.4 (−1.7– −1.2)	−1.2 (−1.5– −1)
Post-exercise	**−3.3 (−3.8– −2.7)** ^†††,‡‡^	^§§^ **−3.1 (−4.7– −1.4)** ^##,¶¶^	−1.5 (−1.8– −1.3)	−1.6 (−2.0– −1.2)
Post-exercise BD	^ƒƒƒ^ −1.2 (−1.5– −0.9)	^ƒƒƒ^ −1.7 (−2.7– −0.7)	^ƒƒƒ^ −0.9 (−1.1– −0.7)	^ƒƒ^ −1.2 (−1.5– −0.9)
**Δ*X*_mean_ (hPa·s·L^−1^)**				
Baseline	−1.0 (−1.6– −0.4)	−0.4 (−0.7– −0.2)	−0.4 (−0.6– −0.3)	−0.5 (−0.7– −0.2)
Post-exercise	^§§^−1.6 (−2.6– −0.7)**^,††,‡‡^	0.0 (−0.7–0.6)	−0.5 (−0.7– −0.3)	−0.5 (−0.7– −0.2)
Post-exercise BD	−1.2 (−2.0– −0.4)	−0.4 (−0.8–0.0)	−0.5 (−0.7– −0.3)	−0.6 (−0.8– −0.4)

Data are presented as mean (95% confidence interval) for continuous data (two-way ANOVA with Bonferroni correction). *R*: resistance; *X*: reactance; E: expiration; I: inspiration; Δ: difference. Significance symbols for bold data values: *: POLD *versus* pPRISm; ^†^: POLD *versus* PT_c_; ^‡^: POLD *versus* T_c_; ^#^: pPRISm *versus* PT_c_; ^¶^: PRISm *versus* T_c_; ^+^: PT_c_ *versus* T_c_. Significance symbols for underlined data values: ^§^: baseline *versus* post-exercise; ^ƒ^: post-exercise *versus* post-BD. Single symbol: p<0.05; double symbol: p<0.01; triple symbol: p<0.001.

*X*_meanE_ and *X*_meanI_ were both significantly more negative in the POLD group when compared to the PT_c_ and T_c_ groups. For the POLD group, there was a more negative Δ*X*_mean_ following exercise (−1.0 to −1.6 hPa·s·L^−1^), while the pPRISm group had more negative reactance (−2.0 to −3.1 hPa·s·L^−1^) following exercise. Almost all groups showed improvement following bronchodilation for both *X*_meanE_ and *X*_meanI_.

## Discussion

We have used intra-breath oscillometry to assess potential differences between phenotypes of PLD, with regard to changes occurring throughout the respiratory cycle, something that cannot be identified with standard oscillometry.

We have demonstrated that children with POLD have impaired impedance throughout the respiratory cycle, particularly in comparison to preterm-born children without any current lung dysfunction and term-born children. This includes increased resistance and more negative reactance during inspiration and expiration, as well as at the end of each phase of the respiratory cycle. Of interest, in children with POLD, mean expiratory impedance (both resistance and reactance) increased in greater magnitude compared to mean inspiratory impedance following exercise. During standard oscillometry there were few differences noted in resistance parameters following exercise [1]. This suggests that exercise affects expiratory flow to a greater degree than inspiratory flow in peripheral airway obstruction in preterm-born children and that intra-breath oscillometry is sensitive for detecting such changes that may not be seen overall using standard oscillometry.

Of specific interest is what happens to impedance at the zero-flow states of end-expiration and end-inspiration, removing potential dynamic factors such as upper airway obstruction [27]. Δ*R* and Δ*X* at baseline showed no significant differences between groups, perhaps unexpectedly for the children with POLD as obstructive lung disease potentially shows increased Δ*R* in particular, such as in the case of preschool wheeze [2] and adult asthma [7], the latter showing that intra-breath oscillometry has greater sensitivity for detecting differences compared to traditional oscillometry. It also differs from the expiratory *versus* inspiratory difference in reactance seen in patients with COPD, potentially associated with flow imitation and linked to dyspnoea [28]. Instead, a pan-respiratory cycle difference was noted in the POLD children suggesting a different pathology to preschool wheeze/asthma phenotypes where the expiratory rather the inspiratory components are most affected.

One possibility is that the nature of the obstructive lung disease is different to these other conditions. Resistance is volume dependent, and children with POLD demonstrate higher functional residual capacity (FRC) compared to controls and children with pPRISm [13]. The combination of higher baseline FRC from air trapping, negating any changes in end-expiratory constrictor tone as seen in other obstructive airway disease [2], plus smaller tidal volumes, thus results in smaller Δ*R* and Δ*X* compared to other obstructive lung disease. Interestingly, when accounting for change in tidal volume in oscillometry, the children with POLD then demonstrated approximately twice the magnitude of Δ*R* compared to both control groups, which suggests that this diminished tidal volume is a significant factor. Furthermore, standard oscillometry has shown that resistance in children with POLD is frequency dependent, with lower frequencies demonstrating higher resistance, suggestive of peripheral airways being affected to the greatest extent. It may be that intra-breath oscillometry performed at 6 Hz would theoretically detect greater differences compared to the higher frequency of 10 Hz we used in this study. However, lower oscillation frequencies are likely to provide worse temporal resolution and are more likely to be contaminated by breathing harmonics, especially given the higher breathing rates of children, which may confound any benefits of using a lower frequency.

Similarly, reactance demonstrated an overall greater negative magnitude at both zero-flow states (*X*_eE_ and *X*_eI_) and throughout the respiratory cycles (*X*_meanE_ and *X*_meanI_) in children with POLD. This is again in keeping with the findings from standard oscillometry where reactance in an obstructive phenotype was more negative overall. Expiratory reactance, including at end-expiration, was more negative than during inspiration. Δ*X* was not significantly different in obstructive or pPRISm phenotypes. However, when normalised against tidal volume, baseline Δ*X* in children with POLD was significantly different compared to both control groups, with a greater difference observed. At 10 Hz, with a negative reactance, compliance is the dominant force. As children with obstructive airway disease most likely have reduced compliance as a result of possible fixed structural defects either within or, more likely, outside of the airways, reactance is disproportionately affected over resistance when tidal volume changes occur, thus greater differences were observed.

Exercise showed little difference for intra-breath values of resistance. This could be for at least two reasons. One relates to the timing of exercise-induced bronchoconstriction, and whether timings of exercise-induced bronchoconstriction detectable with oscillometry, either standard or intra-breath format, are at their peak at 20 min following exercise as noted with spirometry [12]. The other factor as discussed previously is the peripheral location of lung pathology. Potentially there are some differences in how airways in children with POLD and pPRISm respond to exercise, with obstructive phenotypes having typical expiratory changes compared to children with pPRISm, with the latter likely to reflect a degree of restrictive airway, where in inspiration there is perhaps a difference in dynamic compliance of their airways, as demonstrated by the trend towards post-exercise reactance changes seen in inspiration for the pPRISm group and expiration for the POLD group.

Post-exercise bronchodilation generally showed improvements in all groups during all aspects of the respiratory cycle. The greatest responses in Δ*R* and Δ*X* (both in isolation and when accounting for tidal volume changes) were noted following post-exercise bronchodilation in children with POLD. This suggests that while there may not be as clear a distinction as seen in other obstructive lung disease [2], there is still reversible airway obstruction that can be treated with β_2_-agonists which have detectable oscillometry changes. Post-exercise increases in areas within resistance/reactance–volume and reactance–flow curves are seen in children with POLD, which potentially suggests some gas trapping and lung hyperinflation. The influence of subsequent bronchodilation may be to reduce this dynamic hyperinflation, represented by the aforementioned improvements in Δ*R*/*V*_T_ and Δ*X*/*V*_T_.

Children with pPRISm represent another phenotype of PLD which has been underexplored until recently [13, 15]. While this phenotype is likely of interest due to its potential for morbidity, particularly if the changes persist into adulthood, the oscillometry findings are relatively unremarkable, albeit with a trend towards greater impedance compared to controls; however, there is not an obvious picture within intra-breath oscillometry that distinguishes them, unlike the children with POLD. Given this phenotype likely represents a number of children with restrictive pattern of lung disease [13], similarities may then be expected with other restrictive lung diseases, where abnormal end-inspiratory reactances can be seen due to increased distension at end-inspiration [29]. Indeed, post-exercise, a more negative *X*_eI_ was found in children with pPRISm, suggesting that there is some tendency towards this pattern.

By identifying specific phenotypes of PLD, our study suggests several avenues to pursue in the future. Since the trajectories of intra-breath oscillometry especially for the different phenotypes we have described are largely unknown, longitudinal studies would aid understanding of these phenotypes [30]. In addition, the technique can be used in the clinic, given its ease of use. However, robust standardisation and generation of accurate reference values for both sexes, at different ages, heights and of different ethnicity are required. The method has potential to assess response to treatment.

The main strength of this study is the assessment of changes in intra-breath oscillometry measures of recently described phenotypes of PLD, as well as assessment after exercise and after post-exercise bronchodilator administration. The main limitation is the small numbers in the POLD and pPRISm groups, although we had sufficient numbers of controls to compare with. Our findings need to be replicated in larger cohorts of children with PLD. We used local relevant preterm- and term-born controls but there is a need for standardised reference values.

In conclusion, there are limited differences between the zero-flow state in preterm-born children with obstructive airway phenotype, suggesting alternative pathology to that seen in other obstructive airway disease, although differences, particularly in reactance, become apparent once changes in tidal volume are taken into consideration. Detection of exercise-induced changes may be more sensitive. Given peripheral airway disease is likely to be the predominant pathology, investigation of intra-breath changes at lower frequency may distinguish POLD from other obstructive diseases such as asthma and COPD.

## Supplementary material

10.1183/23120541.00840-2024.Supp1 **Please note:** supplementary material is not edited by the Editorial Office, and is uploaded as it has been supplied by the author.
Supplementary material 00840-2024.SUPPLEMENT
