# Supplementary material for: Intra-breath respiratory mechanics of prematurity-associated lung disease phenotypes in school-aged children
Source: ERJ Open Res. 2025 Mar 31;11(2):00840-2024. doi: 10.1183/23120541.00840-2024 (PMC11955913; doi:10.1183/23120541.00840-2024)
Supplement: Supplementary file 1 [file 00840-2024.SUPPLEMENT.pdf]

Intra-breath respiratory mechanics of prematurity-associated lung disease phenotypes in school-aged children

[1,2]Michael Cousins MRCPCH, PhD, [1,2]Kylie Hart PhD, [3]Bence Radics MD, PhD, [4]A John Henderson FRCPCH, PhD, [5]Zoltán Hantos PhD DSc, [6]Peter D Sly MD DSc, [1]Sailesh Kotecha FRCPCH, PhD.

[1]Department of Child Health, Cardiff University School of Medicine, Cardiff, United Kingdom.

[2]Department of Paediatrics, Cardiff and Vale University Health Board, Cardiff, United Kingdom.

[3]Department of Pathology, University of Szeged, Szeged, Hungary.

[4]MRC Integrative Epidemiology Unit, Population Health Sciences, Bristol Medical School, University of Bristol, Bristol, United Kingdom.

[5]Department of Anesthesiology and Intensive Therapy, Semmelweis University, Budapest, Hungary.

[6]Child Health Research Centre, The University of Queensland, South Brisbane, Australia.

**This publication is dedicated to our very dear, late friend Professor John Henderson.

Corresponding Author: Professor Sailesh Kotecha

Department of Child Health

Cardiff University School of Medicine

Cardiff CF14 4XN

United Kingdom

Email: KotechaS@cardiff.ac.uk

Methods

Population

Children were recruited from the Respiratory Health outcomes in NeOnates (RHiNO) study (EudraCT: 2015-003712-20). Inclusion criteria for preterm-born children into the study were birth at ≤34 weeks' gestation, aged 7-12 years, and being geographically accessible. Exclusion criteria were congenital or cardiorespiratory abnormalities, or significant neurodevelopmental impairment. The main RHiNO trial was a randomised control trial comparing a treatment course of inhaled corticosteroids alone or in combination with long-acting beta-2 agonist vs placebo in preterm-born children with percent predicted forced expiratory volume in 1 second (%FEV_1) ≤85%, to assess potential improvement in lung function. The aims of the wider study was to characterise children with lung function decrements based on perinatal, lung function and mechanistic influences, by comparison with preterm- and term-born controls.

Initial screening took place where baseline spirometry (Microloop Spirometer, Vyaire, Germany), along with history exploration and exhaled nitric oxide testing (NiOX VERO, Circassia, UK), was performed [1]. All preterm-born children with %FEV_1 ≤85% at screening were invited for participation in the randomised control trial [2], along with randomly selected preterm-born children with %FEV_1 >85% (within the first 10 screening visits of each calendar month) and all term children with %FEV_1 >90%, for control purposes. In depth lung function testing was performed at the Children and Young Adults' Research Unit at the Noah's Ark Children's Hospital for Wales in Cardiff. Term-born children were only recruited if their %FEV_1 was >90%, therefore no data were obtained from term-born children with lower %FEV_1.

Children were excluded if they were unable to perform adequate spirometry. Children prescribed medication that could potentially affect results were asked to withhold prior to testing for specified time periods [2], and testing delayed in the context of recent respiratory tract infections.

Oscillometry testing

Oscillometry testing was performed using a custom-built set-up and computer programme (NDAQ) developed by a team at University of Szeged in Hungary (Figure E1). A loudspeaker was connected to pressure and flow sensors within a measurement head, at the airway opening. The loudspeaker was further encased within a larger, sealed cylinder connected to above the loudspeaker via a shunt tube for pressure equalisation due to the potential of increased breathing frequency and pressure following exercise.

For testing, children sat upright on a chair, and breathed via a Microgard II microbial filter (Vyaire, Germany). A nose clip was worn and cheeks were held by the child or parent/researcher during testing. The loudspeaker generated a signal at 10Hz with impedance measured at the mouth using the pressure and flow sensors at 100 millisecond intervals. A minimum of 3 recordings over 24 seconds were taken to obtain adequate, artifact-free, sections for analysis.

Intra-breath oscillometry was performed at 3 separate (baseline; 20 minutes following maximal exercise testing; following post-exercise bronchodilation with 400 micrograms of salbutamol (Salamol, TEVA UK Limited) given via MDI using a Volumatic spacer (GSK, UK)).

Raw oscillometry data was analysed post-acquisition to obtain the results. Each of the recordings was assessed, and the trace with most regular, artefact-free respiration was used for analysis, with resistance (Rrs) and reactance (Xrs) measurements at various time points calculated by the software (i.e. at end-inspiration/expiration, maximal flow).

Table E2 displays the parameters analysed.

Spirometry and cardiopulmonary exercise testing (CPET)

Spirometry and cardiopulmonary exercise testing has been described in detail elsewhere [3]. Spirometry was performed using the MasterScreen Body and PFT systems with SentrySuite measurement software version 2.17 (Vyaire Medical, Germany) as per ERS/ATS guidance [4], with a minimum of 3 tests performed, and QC to ensure the appropriate results from all the measurements were used. Calibration was performed as recommended. Results were measured at BTSP and Global Lung Initiative predicted values were used to ensure results comparable [5].

Cardiopulmonary exercise testing was performed on a Pediatric Cycle Ergometer (Lode, Netherlands) linked to a Masterscreen CPX system (Vyaire Medical, Germany). A ramp protocol of increasing Wattage (1 Watt every 6 seconds) following baseline measurements, was used, with testing ending when cadence was no longer consistently maintained. A 'maximal' test was defined by meeting ≥2/4 of the following criteria: Respiratory Exchange Ratio >1.00; heart rate ≥80% predicted (220 bpm – age); ≥9/10 on OMNI scale (pictorial scale for rating of perceived exertion [6]); VO_2 plateau based on visual analysis.

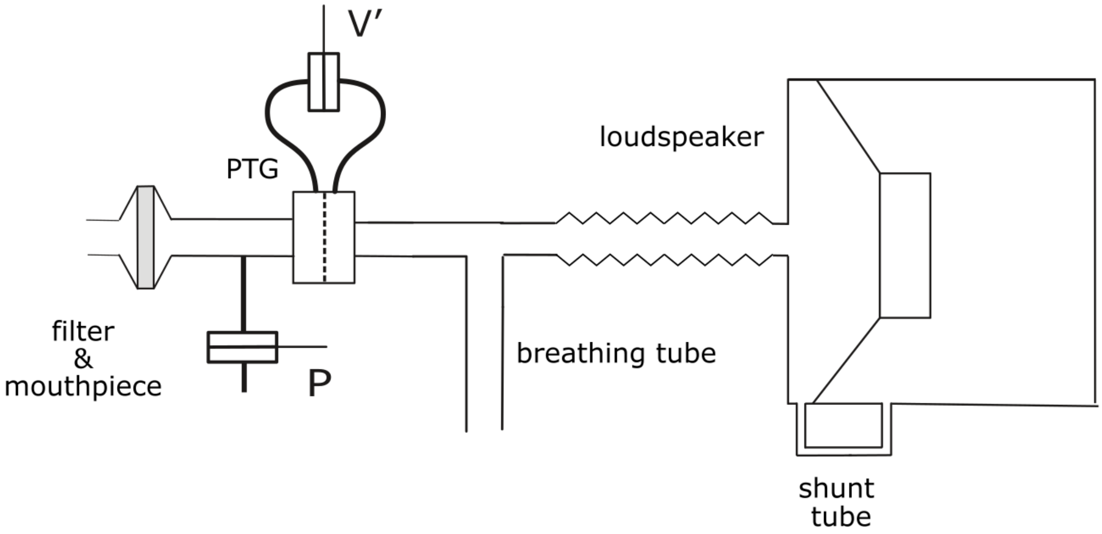

Figure E1. Schematic arrangement of the oscillometry device. PTG: pneumotachograph; pressure (P) and flow (V') sensors: Honeywell model 26PCAFA6D (Golden Valley, MN, USA). Antibacterial filter with mouthpiece (Microgard-II microbial filter, Vyaire, Germany).

Group	Abbreviation	Definition
Prematurity-associated obstructive lung disease	POLD	FEV_1 <LLN; FEV_1/FVC ratio <LLN)
Prematurity-associated preserved ratio of impaired spirometry	pPRISm	(FEV_1 <LLN; FEV_1/FVC ratio ≥LLN)
Preterm Controls	PT_c	FEV_1 ≥LLN
Term Controls	T_c	%FEV_1 >90%

Table E1. Abbreviations and definitions for grouping participants based on lung function.

Parameter	Definition
R	Resistance
X	Reactance
eE<br>eI	Impedance at end expiration/inspiration
ΔR<br>ΔX	Difference in resistance/reactance between end expiration and end inspiration
ΔR/VT<br>ΔX/VT	Difference in resistance/reactance between end expiration and end inspiration, adjusted for tidal volume
meanE/I	Mean impedance in expiration/inspiration
ARV<br>AXV	Area within the resistance/reactance-volume loops
ARV'<br>AXV'	Area within the resistance/reactance-flow loops

Table E2. Intra-breath parameters used in analysis with abbreviations and explanations.

References

1. Hart K, Cousins M, Watkins WJ, et al. Association of Early Life Factors with Prematurity-Associated Lung Disease: Prospective Cohort Study. Eur Respir J 2021: 2101766.
2. Goulden N, Cousins M, Hart K, et al. Inhaled corticosteroids alone and in combination with long-acting β-2 receptor agonists to treat reduced lung function in preterm-born children; A randomized clinical trial. JAMA Pediatr 2021; 176(2): 133-141.
3. Cousins M, Hart K, Williams EM, et al. Impaired exercise outcomes with significant bronchodilator responsiveness in children with prematurity-associated obstructive lung disease. Pediatr Pulmonol 2022(57): 2161-2171.
4. Miller MR, Hankinson J, Brusasco V, et al. Standardisation of spirometry. Eur Respir J 2005; 26(2): 319–338.
5. Quanjer PH, Stanojevic S, Cole TJ, et al. Multi-ethnic reference values for spirometry for the 3-95-yr age range: the global lung function 2012 equations. Eur Respir J 2012; 40(6): 1324–1343.
6. Barkley JE, Roemmich JN. Validity of the CALER and OMNI-Bike Ratings of Perceived Exertion. Med Sci Sports Exerc 2008; 40(4): 760-766.
